# Editorial: Recent advances in mitochondria-associated endoplasmic reticulum membranes (MAMs) in heart-related diseases: volume II

**DOI:** 10.3389/fcvm.2025.1751239

**Published:** 2025-12-15

**Authors:** Ziheng Zhang, Kaidi Ren, Yang Yang

**Affiliations:** 1 College of Life Science and Technology, Xinjiang University, Urumqi, China; 2 Department of Pharmacy, The First Affiliated Hospital of Zhengzhou University, Zhengzhou, China; 3 Clinical Systems Biology Key Laboratories, The First Affiliated Hospital of Zhengzhou University, Zhengzhou, China

**Keywords:** mitochondria, endoplasmic reticulum, mitochondria-associated endoplasmic reticulum membranes, heart, heart-related diseases

A deeper mechanistic understanding of cardiac biology increasingly reveals that cardiomyocyte homeostasis is maintained not by the autonomous functioning of individual organelles but through an intricate network of physical and biochemical interactions. Within this network, the interface between mitochondria and the endoplasmic reticulum has emerged as a central coordinator of metabolic flux, calcium exchange, redox balance, and structural remodeling (1). These contact sites, often referred to as mitochondria-associated ER membranes, function as highly specialized microdomains capable of rapidly integrating metabolic demand with stress signaling. In a tissue that is metabolically intensive and rhythmically constrained such as the heart, even subtle disturbances in these inter-organelle circuits can precipitate significant physiological derangements, impairing excitation‒contraction coupling, undermining ATP generation, and increasing susceptibility to cell death (2, 3). Recent advances in imaging, proteomics, and cellular modeling have begun to illuminate the dynamic plasticity of these interfaces, positioning mitochondrial-ER communication as a foundational determinant of cardiac resilience.

This shift from an organelle-centric view to a communication-centric perspective reframes cardiac pathology as a disorder of intracellular coordination. Mitochondria-associated ER membranes, once considered peripheral structural features, are now understood as adaptive platforms capable of reorganizing in response to metabolic shifts, mechanical loads, or inflammatory stress (4, 5). Perturbations at these junctions propagate across a broader landscape of intracellular regulation, influencing autophagic flux, protein synthesis fidelity, lipid storage and utilization, the mitochondrial fission‒fusion balance, and even nuclear transcriptional programs (5). This systems-level architecture provides a unifying framework through which diverse pathological triggers, ranging from inherited metabolic defects to chronic neurohormonal stimulation, converge on common mechanistic pathways that dictate cardiomyocyte fate (6, 7). Emerging evidence situates organelle communication not as an epiphenomenon of disease but as a core axis of cardiac adaptation and degeneration.

Increasing attention has also focused on the fact that organelle communication operates as an adaptive regulatory system with its own spatial logic and temporal cadence rather than a static set of molecular contacts. In the constantly fluctuating mechanical and metabolic environment of the heart, mitochondria‒ER interfaces act as real-time integrators that recalibrate calcium transients, redox gradients, and biosynthetic outputs with remarkable precision (1). Their architecture is continuously remodeled in response to workload, substrate availability, inflammatory cues, and neurohormonal stimulation, allowing cardiomyocytes to preserve functional equilibrium under diverse forms of stress. When this plasticity is blunted through aberrant expression of tethering proteins, disruption of membrane lipid organization, or sustained mechanical strain, the same contact sites that normally sustain homeostasis can redirect signaling toward maladaptive pathways. This framework underscores that the pathological shift is not merely a consequence of organelle dysfunction but rather a failure in the dynamic rules that govern how intracellular communication networks adapt, reorganize, and ultimately determine cardiomyocyte fate (8, 9).

Against this conceptual backdrop, the studies included in this research collectively refine our understanding of how inter-organelle signaling contributes to the development and progression of cardiovascular disorders. The role of mitochondria-lysosome interplay is illustrated in infantile-onset Pompe disease, where disruption of lysosomal glycogen clearance destabilizes mitochondrial integrity, impairs autophagic degradation, and amplifies oxidative injury, thereby aggravating metabolic cardiomyopathy (Zhang et al.). Extending the focus to electrophysiological stability, research employing human iPSC-derived cardiomyocytes has demonstrated how β-adrenergic agonists differentially shape beating behavior and arrhythmic liability, providing mechanistic insight into calcium-handling disturbances tightly linked to mitochondrial–ER coordination (Yuan et al.). Complementary perspectives arise from the synthesis of mitochondrial communication with the ER, lysosomes, ribosomes, lipid droplets, and nucleus, revealing how the collapse of these integrated networks accelerates heart failure through defects in proteostasis, metabolic signaling, and adaptive stress responses (Chang et al.). Parallel work examining unfolded protein response pathways across multiple cardiomyopathy subtypes highlights the dual nature of ER stress, which is initially protective yet ultimately deleterious when it is chronically activated, particularly through its intersection with mitochondrial dysfunction and apoptotic pathways (Qiu et al.). An analysis of dilated cardiomyopathy further underscores how structural remodeling of ER-mitochondria interfaces disrupts calcium flux, lipid coordination, mitochondrial morphodynamics, and inflammatory signaling, collectively driving contractile decline and chamber dilation (He et al.). Finally, a focused examination of mitofusin-2 synthesizes current knowledge of how this dual-localized tether modulates fusion, calcium microdomains, lipid transfer, and stress signaling while emphasizing the unresolved context-dependence of its cardioprotective vs. maladaptive roles (Lv et al.). Taken together, these findings reveal a common theme: cardiac disease frequently reflects a breakdown in the fidelity of organelle communication, and restoring this intracellular connectivity may represent a powerful therapeutic strategy.

Looking ahead, it is increasingly clear that the future of cardiovascular research hinges on uncovering the rules that govern the spatial and temporal dynamics of inter-organelle interactions. Key unanswered questions include how contact sites reorganize in response to acute vs. chronic stress, how metabolic cues influence tethering protein turnover, and how pathologic transitions alter the topology of intracellular communication networks. The integration of single-cell multi-omics, spatial proteomics, live-cell super-resolution imaging, and computational reconstruction of organelle interactomes holds immense promise for mapping these dynamics with unprecedented precision. These approaches will likely reveal previously unrecognized microdomains of signaling exchange and identify novel molecular determinants that govern the conversion of reversible adaptation into irreversible injury.

From a translational perspective, the challenge is to design interventions that modulate organelle communication with specificity and nuance. Blanket augmentation or suppression of contact site formation is unlikely to yield safe or durable benefits, given the essential physiological roles of these domains. Instead, future therapies may target discrete tethering complexes, post-translational modifiers, or metabolite-sensitive microdomains that selectively orchestrate maladaptive signaling. Advances in gene-editing strategies, organelle-targeted delivery systems, and engineered peptides open the possibility of correcting pathogenic remodeling of tethering proteins or rebalancing calcium and lipid flux without disrupting global cellular function. By shifting from downstream symptom management to upstream restoration of the intracellular communication architecture, the field may move closer to disease-modifying treatments capable of altering the natural history of heart failure and cardiomyopathy.
